# A Time-Based Electronic Front-End for a Capacitive Particle Matter Detector

**DOI:** 10.3390/s21051840

**Published:** 2021-03-06

**Authors:** Umberto Ferlito, Alfio Dario Grasso, Michele Vaiana, Giuseppe Bruno

**Affiliations:** 1 Dipartimento di Ingegneria Elettrica Elettronica e Informatica (DIEEI), University of Catania, 95125 Catania, Italy; umberto.ferlito@unict.it; 2 STMicroelectronics, 95121 Catania, Italy; michele.vaiana@st.com (M.V.); giuseppe.bruno@st.com (G.B.)

**Keywords:** capacitive sensor, particulate matter (PM), ring oscillators, smart sensors

## Abstract

This paper introduces the electronic interface for a capacitive airborne particle matter detector. The proposed circuit relies on two matched ring oscillators and a mixer to detect the frequency difference induced by the deposition of a particle onto an interdigitated capacitor, which constitutes the load of one of the oscillators. The output of the mixer is digitized through a simple counter. In order to compensate the oscillation frequency offset of the two ring oscillators due to process and mismatch variations, a capacitive trimming circuit has been implemented. The sensor is connected to host through an I2C interface for communication and configuration. The sensor has been implemented using a standard 130-nm CMOS technology by STMicroelectronics and occupies 0.12-mm^2^ die area. Experimental measurements using talcum powder show a sensitivity of 60 kHz/fF and a 3σ resolution equal to 165 aF.

## 1. Introduction

Atmospheric particle matter (PM) is a category of airborne pollutants that includes dust, tobacco smoke, diesel exhaust, and other primary sources. Fine particles that have a diameter between 10 μm (PM_10_) and 2.5 μm (PM_2.5_) represent a threat for human health because of their ability to penetrate deep into the respiratory system. Indeed, exposure to PM_10_ and PM_2.5_ has been linked to a reduction of the life expectancy between 8 and 36 months [1,2,3,4,5,6,7,8,9].

Conventional methods to monitor PM concentration are based on gravimetric or laser scattering detection methods that are bulky, costly, and do not allow appropriate spatiotemporal resolution. With the aim of reducing the sensor volume and enable ubiquitous PM measurement, solutions exploiting high-resolution capacitive sensors have been recently proposed [1,2,4,6,9]. As well known, capacitance detection represents a universal transduction mechanism, leveraged in a large variety of sensors and applications, thanks to the smartness and the conceptual simplicity [10], [11].

Capacitive PM detection represents, however, a challenging task due to the small capacitance variation induced by a single particle. Indeed, a particle with a radius between 1.25 µm and 20 µm induces over a planar capacitor a capacitance variation in the order of tens attofarad. [3,4,5]. However, in the case of a parallel and planar faces capacitor, which requires additional steps during the fabrication process, the variations would be an order magnitude bigger [1,12].

The electronic front-end therefore plays a fundamental role because it defines the ultimate resolution limit, power consumption, and area occupation of the overall sensor. Given the importance and widespread diffusion of integrated capacitive sensors, it is quite understandable that many different electronic interface topologies have been presented in literature, such as capacitance-to-voltage (C2V) [1,2,3,4,5,10,13,14,15,16,17,18], charge based capacitance measurement (CBCM) [19,20,21,22,23,24,25,26,27], capacitance-to-current (C2I) [28,29,30], capacitance-to-time (C2T) [31,32,33,34,35], and capacitance to digital (C2D) [36,37,38,39,40,41,42]. Each method presents very different features in terms of resolution, area occupation, circuit, complexity, measuring range, and measuring time. Consequently, the adoption of a specific topology depends upon the constraints given by the particular application [11]. As an example, among the solutions in literature, only that reported in [4], which is based on C2V lock-in topology, shows a value of the resolution as low as low as 65 zF and targets PM detection. However, this is achieved at the expense of high-power consumption, area occupation, and partial on-chip implementation [11]. Regarding area occupation, solution in [29] based on C2I topology achieves a very low value of occupied area (0.03 mm^2^), but this comes at the expense of worse resolution (800 aF). Similar conclusions can be carried out for all the other topologies [11]. However, a key aspect to consider is represented by circuit complexity, especially when several replicas of the same circuit must be implemented to process an array of capacitive transducers. In this case, CBCM and C2T topologies represents a good choice. Another aspect to consider when selecting a particular topology is represented by the availability of a digital output. Among the various solutions, only C2D topology inherently provides a digital output, although also C2T solution can achieve this feature by exploiting a simple counter. All of the other solutions require additional ADC converters, which increases further area and power consumption.

With the aim of implementing a fully integrated, simple, while effective, electronic front-end for a capacitive-based PM detector, in this paper we propose a circuit topology, based on C2T architecture, to detect the capacitance variation of a planar capacitor due to the deposition of PM. The proposed solution is based on the calculation of a frequency difference between two identical and matched ring oscillators, where the reference one exhibits always the same oscillating frequency, and the second one exhibits a varying oscillating frequency due to the variation of the permittivity of the sensing capacitor. A digital output is obtained by exploiting a counter and an I2C interface, which is also exploited to trim the frequency of the ring oscillators.

The remainder of this paper is organized as follows. Analysis, design, and simulation results of the proposed system are reported in Section 2. Section 3 reports the experimental measurements and a comparison with the state of the art. Finally, concluding remarks are drawn in Section 4.

## 2. Electronic Front-End Design

**Figure 1:**
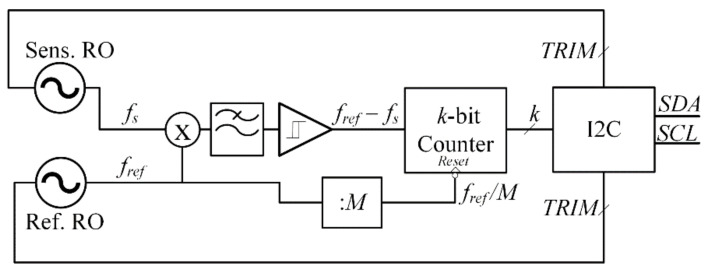
Block diagram of the proposed circuit.

The simplified block diagram of the electronic front end is shown in Figure 1. It is composed by a reference ring oscillator (RO) and another RO whose frequency changes according to the capacitance variation induced by the deposition of PM on sensing capacitors connected at the output of each stage. The frequency difference between the two oscillators is detected through a mixer and a low-pass filter. Finally, a Schmitt trigger and a counter are exploited to get a digital binary word whose value is proportional to the capacitance variation.

The core of the proposed system in Figure 1 exploits a similar principle of operation as that introduced in [32], where, however, the reference and sensing signals are processed by an XOR gate, which generates a signal whose frequency is the maximum common divider of the two input frequencies, which can assume the same value for different combinations of *f_s_* and *f_ref_*. This drawback is overcome using the mixer in the proposed system.

### 2.1. Description and Analysis of the Main Building Blocks

**Figure 2:**
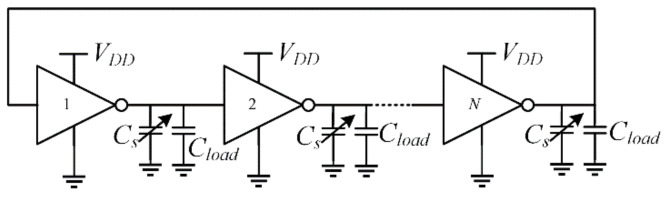
Simplified schematic of the sensing oscillator (reference oscillator has the same topology but with *C_s_* = *C_r_*).

The schematic of each RO is shown in Figure 2. The load capacitor of each stage is made up by an interdigitated integrated capacitor, *C_S_*, which is exploited to detect the particles. An equal capacitor is used for the reference oscillator, but this is isolated from the environment by means of an oxide. Capacitor *C_load_* is equal to *C_S0_* + *C_PAR_* + C*_TRIM_*, being *C_S_*_0_ the nominal value of the sensing capacitance, *C_PAR_* the intrinsic parasitic capacitance of the inverter, and *C_TRIM_* the absolute capacitance of the trimming block, which is described in detail in Section 2.3.

Assuming that the two ROs share the same power supply, the oscillation frequency of the sensing and reference RO is respectively given by

(1) $$f_{s}=\frac{2I_{avg,s}}{V_{DD}\left(NC_{load}+\sum_{i=1}^{N}\Delta C_{i}\right)}$$

(2) $$f_{ref}=\frac{2I_{avg,ref}}{NV_{DD}C_{load}}$$

where *I_avg,s_* and *I_avg,ref_* is the average current of each stage of the sensing and reference RO, respectively, *V_DD_* is the supply voltage, and Δ*C_i_* represents the capacitance variation of the *i*-th stage sensing capacitor of the sensing RO.

**Figure 3:**
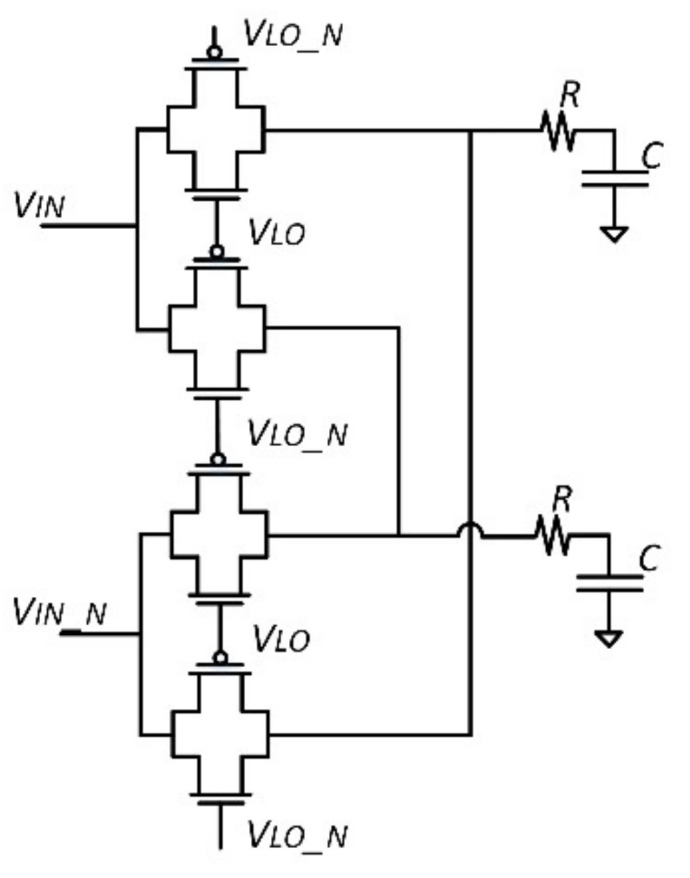
Schematic of the passive mixer and the low-pass filter.

**Figure 4:**
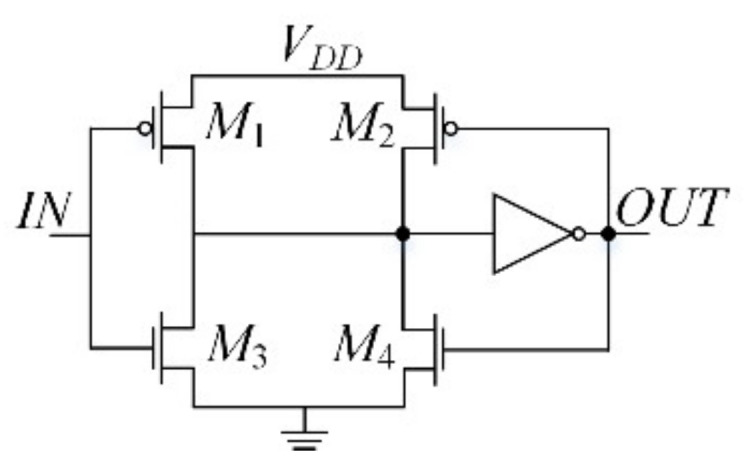
Schematic of the Schmitt trigger.

A passive *RC* low-pass filter is used at the output of a passive mixer Figure 3 to eliminate high frequency components. After that, a Schmitt trigger Figure 4 converts the signal to a square wave, which is finally fed to a binary counter. Assuming ideal operation of the mixer, the low-pass filter, and the Schmitt trigger, the frequency difference of the signal at the input of the counter due to a total capacitance variation (Note that the capacitance variations at the output of each stage are in general different from each other.) is expressed, using Equation (1), by

(3) $$\Delta f=f_{ref}-f_{s}=\frac{2I_{avg}}{V_{DD}}\frac{\sum_{i=1}^{N}\Delta C_{i}}{NC_{load}\left(NC_{load}+\sum_{i=1}^{N}\Delta C_{i}\right)}\approx \frac{2I_{avg}}{V_{DD}}\frac{\sum_{i=1}^{N}\Delta C_{i}}{{\left(NC_{load}\right)}^{2}}$$

where it is assumed that the average current is equal for all the stages. The rightmost approximation in Equation (3) holds assuming small capacitance variations (Although Equation (3) shows a hyperbolic dependence on Δ*C*, it can be approximated with its series Taylor expansion truncated to the first term which is equal to the rightmost term of (3)). From Equation (3), it is apparent that the frequency difference is increased when *C_load_* and *N* are decreased (i.e., when the nominal oscillation frequency is maximized). This can be achieved by reducing the parasitic capacitance of the inverters, by reducing the nominal value of the sensing capacitor and, in particular, by adopting the minimum number of stages.

The number of counts at the output of the counter can be simply obtained by multiplying the frequency of the filter by the reset period of the counter, thus yielding

(4) $$Counts=\Delta f\frac{M}{f_{ref}}$$

From Equation (4), it apparent that the counts are proportional to the ratio between Δ*f* and *f_ref_* and, consequently, the dependence on supply voltage and average current is eliminated.

Assuming a minimum and maximum detectable frequency variation equal to Δ*f_max_* and Δ*f_min_*, respectively, the number of bits of the counter is given by

(5) $$K=\log_{2}\left(\frac{\Delta f_{\max}}{\Delta f_{\min}}\right)$$

Finally, by combining Equations (4) and (5), we obtain the value of the frequency divisor as

(6) $$M=2^{k}\frac{f_{ref}}{\Delta f_{\max}}$$

The minimum detectable capacitance variation is limited by the noise of the various building blocks in Figure 1. A simplified noise analysis can be carried out assuming that all the noise sources are uncorrelated and neglecting the jitter introduced by the mixer, low pass filter and Schmitt trigger. Consequently, the main source of jitter is assumed to be the two ROs. Using results in [43], the total jitter variance of a RO based on CMOS inverters due to white noise is given by

(7) $$\sigma_{}^{2}=\frac{2kT}{I_{avg}f_{0}}\left[\frac{\gamma_{N}+\gamma_{P}}{V_{DD}-V_{th}}+\frac{1}{V_{DD}}\right]$$

where *k* is the Boltzmann constant, *T* is the absolute temperature, *f*_0_ is the nominal oscillation frequency expressed by Equation (1), *V_th_* is the PMOS and NMOS threshold voltage, *γ_N_* and *γ_P_* are the channel noise factors equal to 2/3 in saturation region. The signal-to-noise ratio is then evaluated as

(8) $$SNR=\frac{{\left(\frac{{\left(\Delta f\right)}^{-1}}{\sqrt{2}}\right)}^{2}}{\sigma_{ref}^{2}+\sigma_{s}^{2}}$$

where σ*_ref_* and σ*_s_* is the jitter variance of the reference and sensing RO, respectively.

### 2.2. Simulation Results

The proposed system in Figure 1 has been designed using the 130-nm HCMOS9A technology by STMicroelectronics. The RO are designed assuming *V_DD_* = 1.2 V and, according to Equation (3), setting *N* = 3 to maximize the sensitivity and the *SNR*. The resolution of the counter is set according to Equation (4) with Δ*f_LSB_* = 2.4 kHz and Δ*f_MAX_* = 625 kHz (which corresponds to a maximum detectable capacitance variation of about 10 fF) and is equal to 8 bits. The frequency divisor from Equation (5) is the equal to 2^15^. The other parameters are reported in Table 1, which yields a nominal oscillation frequency equal to 79.9 MHz.

**Figure 5:**
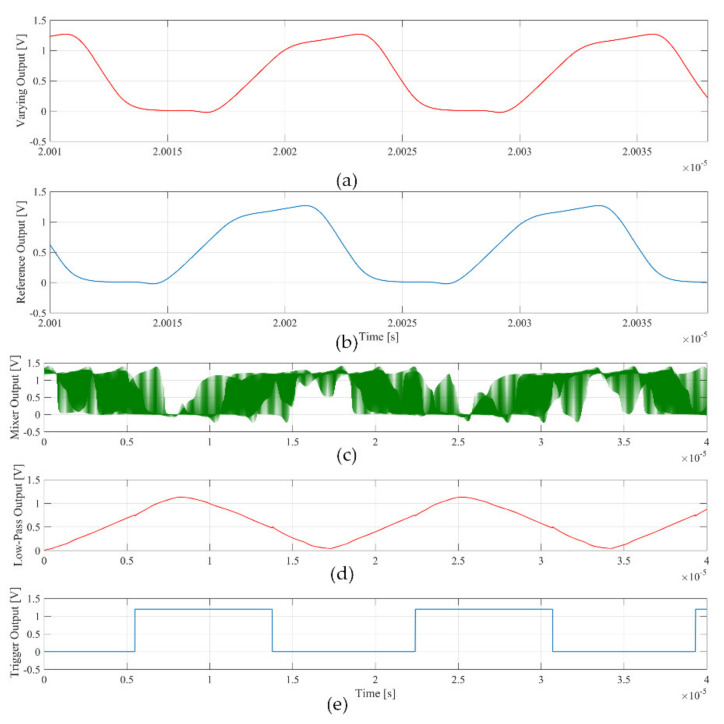
Simulated signals at the output of the blocks in Figure 1: (**a**) sensing oscillator; (**b**) reference oscillator; (**c**) mixer; (**d**) low-pass filter, and (**e**) Schmitt trigger.

Figure 5 shows the post-layout simulation of the signal at the output of the sensing and reference oscillator, the mixer, the low-pass filter and the Schmitt trigger for Δ*C_i_* = 1 fF. The frequency of the signal at the output of the trigger is equal to 60 kHz, as predicted by Equation (3). The simulated noise produces an uncertainty of the measured frequency variation, which determines the minimum detectable capacitance variation. In particular, a *noisetran* simulation over 50 runs with Δ*C_i_* = 1 fF gives a mean frequency variation value that coincides with the value predicted by (2) with Δ*f_RMS_*≈0.8 kHz.

Considering a Gaussian distribution of noise, we should set as minimum resolution 3Δ*f_RMS_*≈2.4 kHz, which corresponds to a 3ΔC*_RMS_* = 40 aF. This value is calculated at the output of the mixer. 

**Figure 6:**
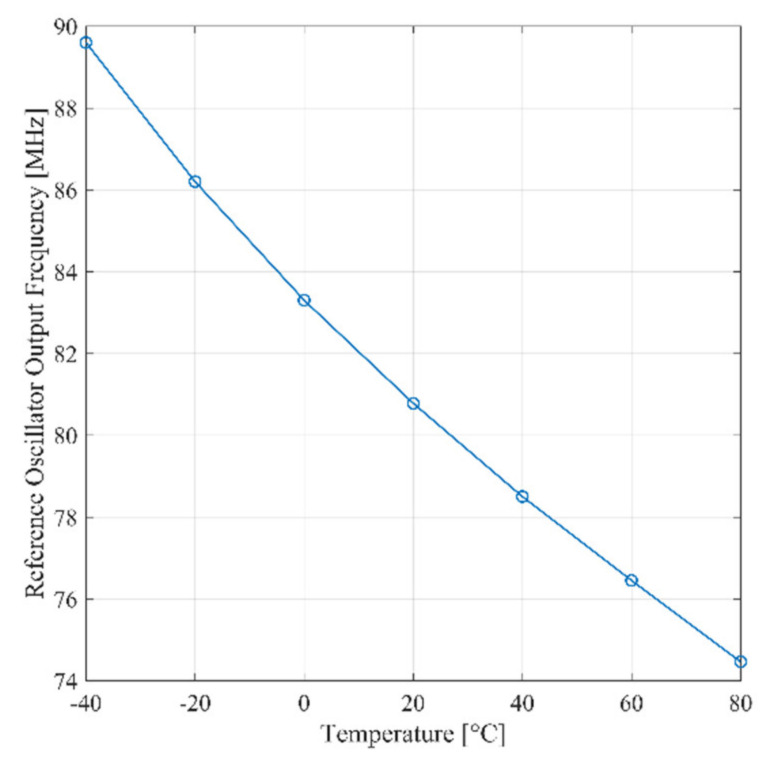
Simulated signal frequency versus temperature for Δ*C_i_* = 1 fF of reference ring oscillator (RO) and Schmitt trigger.

The frequency of the signal at the output of the Schmitt trigger as predicted by Equation (3) is proportional to the average charge current. Consequently, it is expected that the frequency varies with temperature. By inspection of Equation (1), the same conclusion applies for the reference ring oscillator. As a confirmation of these observations, Figure 6 shows the post-layout simulation of the frequency of the signal at the output of reference oscillator and the Schmitt trigger, respectively, versus temperature for Δ*C_i_* = 1 fF. The temperature coefficient of the signal at the output of the trigger over the industrial temperature range (−40:80°) is equal to −108 Hz/°C, while the one of the reference signal is equal to −126 kHz/°C. The simulation results in Figure 6 is coherent with the analytical model carried out in Section II.A, as detailed in Appendix A.

Finally, Table 2 summarizes corner analysis results assuming Δ*C_i_* = 1 fF.

### 2.3. Design of the Capacitive Trimming Circuit

**Figure 7:**
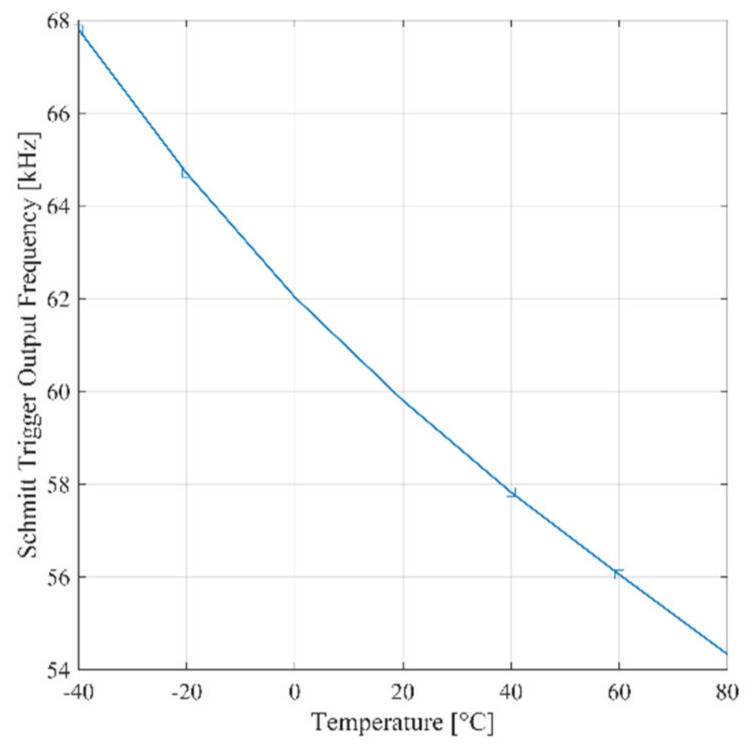
Monte Carlo simulation results with a frequency variation of 245 kHz (Δ*C_i_ =* 4.1 fF).

Mismatch and process variations cause a random offset of the nominal oscillation frequency of the two ROs, which in turn increase the minimum detectable capacitance variation. Monte Carlo simulations in Figure 7 show that, imposing a capacitance variation equal to 4.1 fF, around the nominal output frequency (µ = 246.689 kHz), the standard deviation, σ, is equal to 80 kHz. Thus, we must be able to trim at least 240 kHz (3σ).

To reduce the offset at the output of the system due to process and mismatch variations, a capacitive trimming circuit is adopted to change the *C_TRIM_* load capacitor and re-tune the oscillation frequency to the nominal value. At this purpose, the capacitive trimming circuits must be able to induce a frequency variation equal to about ±3σ = ±240 kHz, or 480 kHz as entire range.

**Figure 8:**
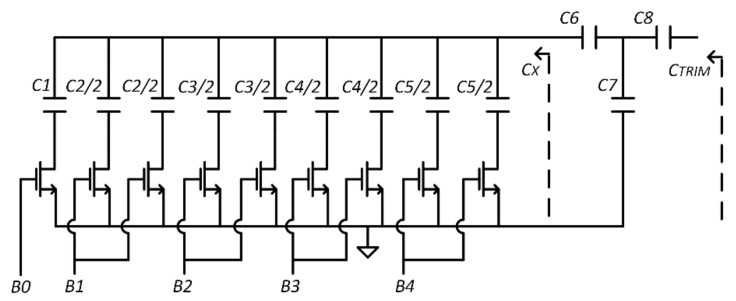
Capacitive trimming circuit schematic.

The schematic of a single capacitive trimming circuit is reported in Figure 8. It is made up by five binary weighted capacitors, *C*_1_–*C*_5_. The minimum capacitor, *C*_1_, is equal to 20 fF. To increase matching, all the other capacitors are obtained by paralleling a proper number of minimum capacitors. Five switches, controlled through the I^2^C interface, are used to get 32 different configurations. Bridge capacitors *C*_6_, *C*_7_, and *C*_8_ are used to reduce the capacitance variation (Considering that the sensitivity is equal to 60 kHz/fF, a variation of 4 fF is required to fully cover the error range of 240 kHz). The equivalent capacitance implemented by the capacitive trimming circuit is expressed by

(9) $$C_{TRIM}=\frac{\left[\frac{\left(C_{X}C_{6}\right)}{\left(C_{X}+C_{6}\right)}+C_{7}\right]C_{8}}{\left[\frac{\left(C_{X}C_{6}\right)}{\left(C_{X}+C_{6}\right)}+C_{7}\right]+C_{8}}$$

where *C_X_* is the equivalent parallel capacitor, which is a function of the switch configuration.

Parasitic capacitances introduced by switches and metal connect can heavily affect trimming since they are commensurable to the capacitance variation to be measured. Consequently, a good layout strategy is mandatory. In order to preserve symmetry, every capacitance in Figure 8 has been divided in two and driven by two equally driven switches. The remaining capacitances have been connected to minimize metallization length and, as usual, adding dummy components to equalize boundary condition. 

The capacitive trimming circuit is replicated also in the reference oscillator to cover the required frequency variation range (±3*σ* = ±240 kHz).

**Figure 9:**
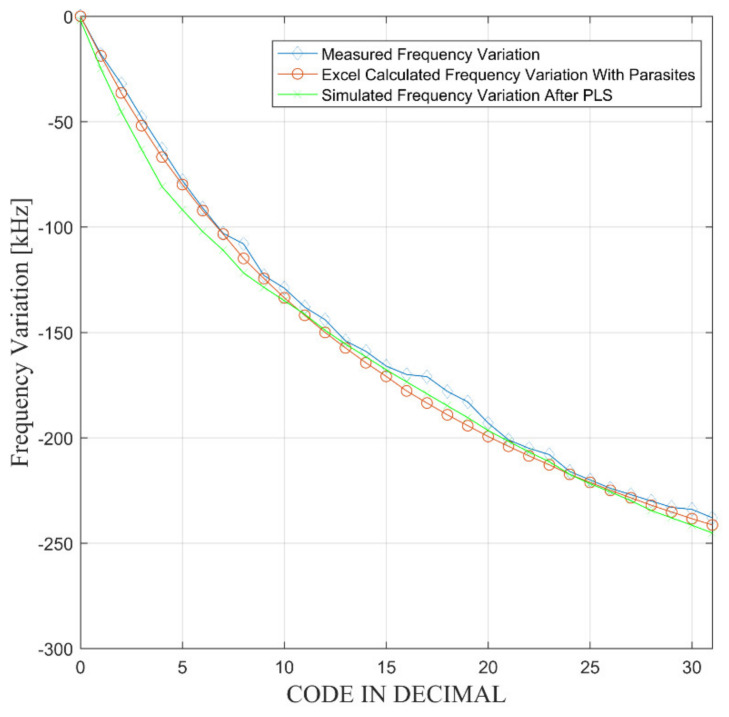
Frequency variation as a function of the binary word.

Figure 9 reports the comparison between the frequency variation predicted by Equation (3) introducing the trimming capacitance Equation (9), the post layout simulations, and the measurement results. It is apparently a good fit between the three curves.

## 3. Measurement Results

**Figure 10:**
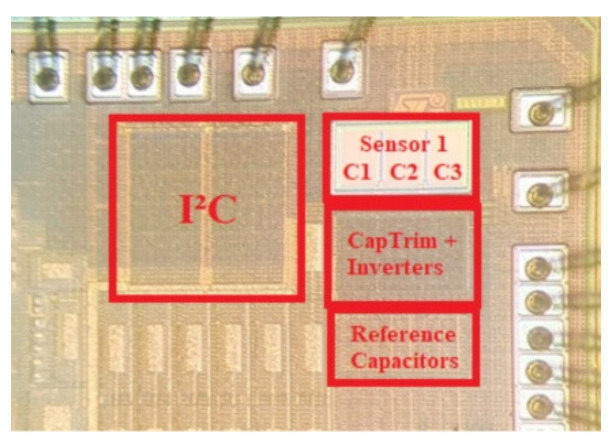
Microphotograph of the chip.

Figure 10 shows the microphotograph of the implemented system. The sensing capacitors are completely exposed through the removal of the passivation and the 200-nm nitride silicon layers. 

The reference capacitor, on the contrary, was passivated. This design choice, however, introduced an additional mismatch between the loading capacitors of the reference and sensing ROs. Consequently, the resulting frequency difference at the output of the Schmitt trigger at standby conditions was only partially compensated by the capacitive trimming circuit because the overall mismatch was higher than the one predicted in simulations. To get rid of the additional mismatch caused by the passivation of the reference capacitor, the oscillators were powered using a different power supply generator.

Total occupied area is 0.12 mm^2^, including front-end, sensor, and reference capacitances. In particular, the electronic front-end occupies 0.06 mm^2^, while the remaining 0.06 mm^2^ are due to the reference and sensing capacitors.

**Figure 11:**
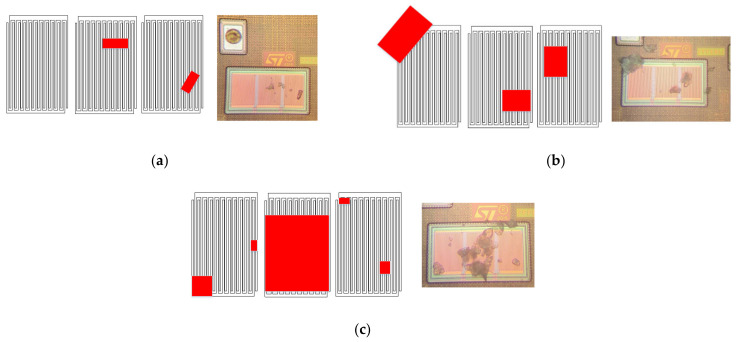
Comparison between the micro photographed particle concentration and the simulation: (**a**) low coverage; (**b**) medium coverage; (**c**) high coverage.

**Figure 12:**
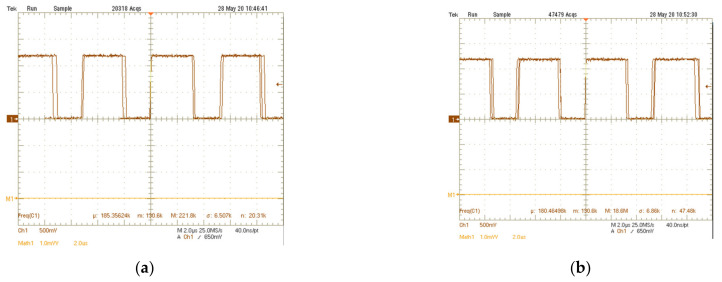

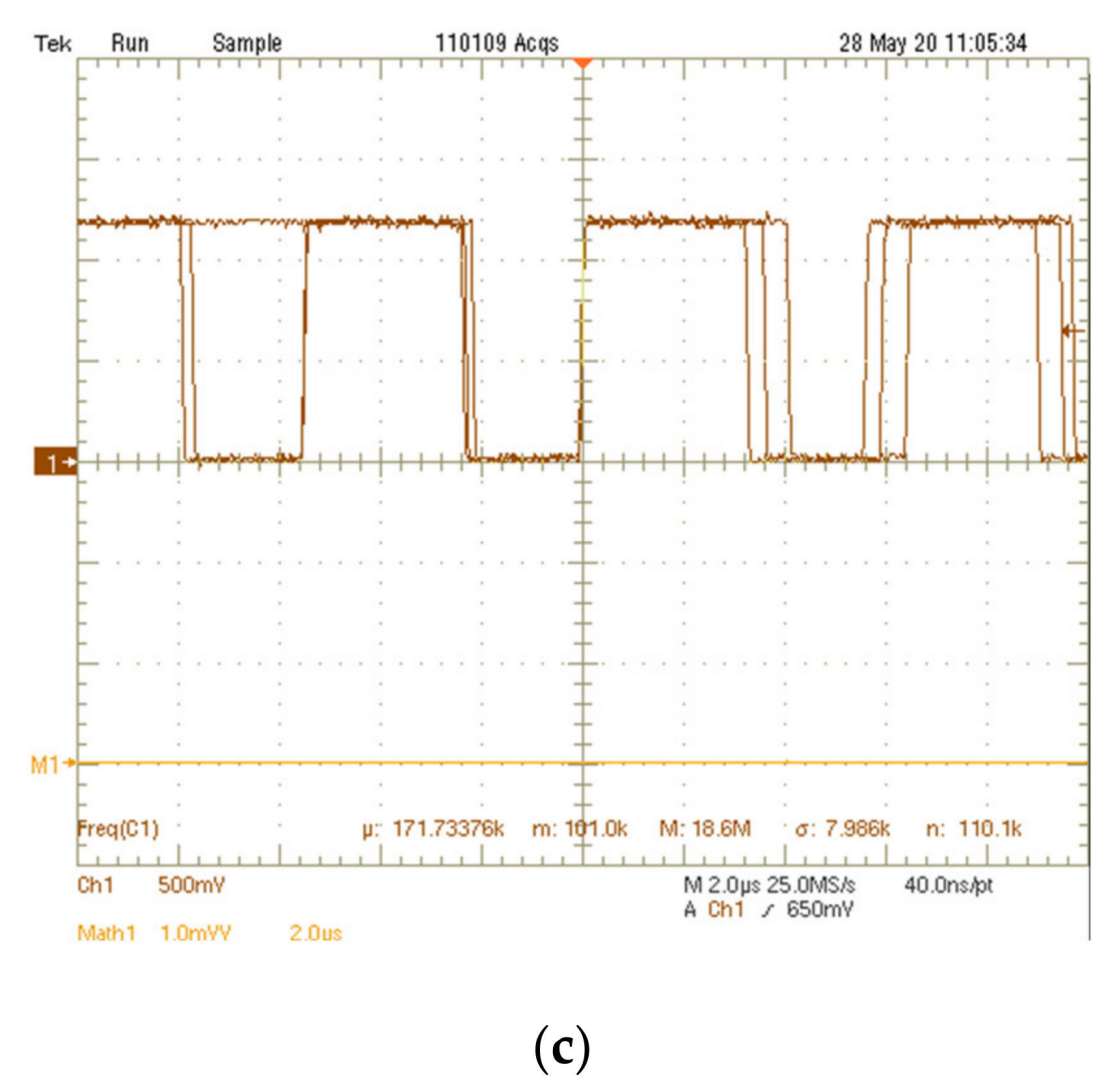
Measured signal at the Schmitt trigger output for the three cases in Figure 11: (**a**) low coverage; (**b**) medium coverage; (**c**) high coverage.

The measurements have been carried out through the deposition of talcum powder particles (ε*_r_* = 2) over the sensing capacitors. During experimental measurements, three main cases have been considered for the electrode coverage, namely low coverage, medium coverage, and high coverage. Figure 11 shows the microphotograph of the electrodes in the three considered conditions. Measured signals at the output of the trigger are reported in Figure 12. In the first case (low coverage), the measured Δ*f* is equal to 5 kHz, which corresponds to a total capacitance variation of Δ*C* = 83 aF. In the second case (medium coverage), Δ*f* = 10 kHz that is related to a total capacitance variation Δ*C* = 166 aF. Finally, in the last case (high coverage) Δ*f* = 18 kHz and Δ*C* = 300 aF.

In order to assess the validity of the measured capacitance variation induced by the talcum deposition, each case has been simulated using COMSOL Multiphysics, as detailed in the same Figure 11. Every capacitor in each case shows a different covering talcum concentration and thus a different capacitance variation. In particular, in the low coverage case, moving from the leftmost pixel to the rightmost one, the variations are respectively 0 aF, 111 aF, and 113 aF. In the medium coverage case, they are 146 aF, 148 aF, and 152 aF and finally, in the high coverage case, they are 114 aF, 1024 aF, and 45 aF. By looking at Equation (3), it is obvious that in case the variations are different, by making the average through the dividing *N* factor, it falls back in case they are equal. By this operation, the equivalent variations are 74 aF, 148 aF, and 394 aF. Note that there is a good matching between the predicted and measured values in the first and second case, where the model better describes the measured case. In the last case, the error between simulation and measurement is higher due to the difficulty of accurate modeling the actual distribution of the particles over the electrodes. Similar measurements were executed on other eight samples with similar results.

The reading error of the sensor is due to electronic noise and to the quantization error introduced by the counter. This error leads to a minimum detectable capacitance, i.e., it determines the resolution. It has been assessed by evaluating the mean value and standard deviation of 20 consecutive readings. The mean value of the hexadecimal word has been multiplied by the resolution of the counter and then compared to the output trigger frequency, resulting in a very good matching.

Table 3 summarizes the measurements executed on the first sample, showing a 3 resolution equal to 165 aF in the worst case.

**Figure 13:**
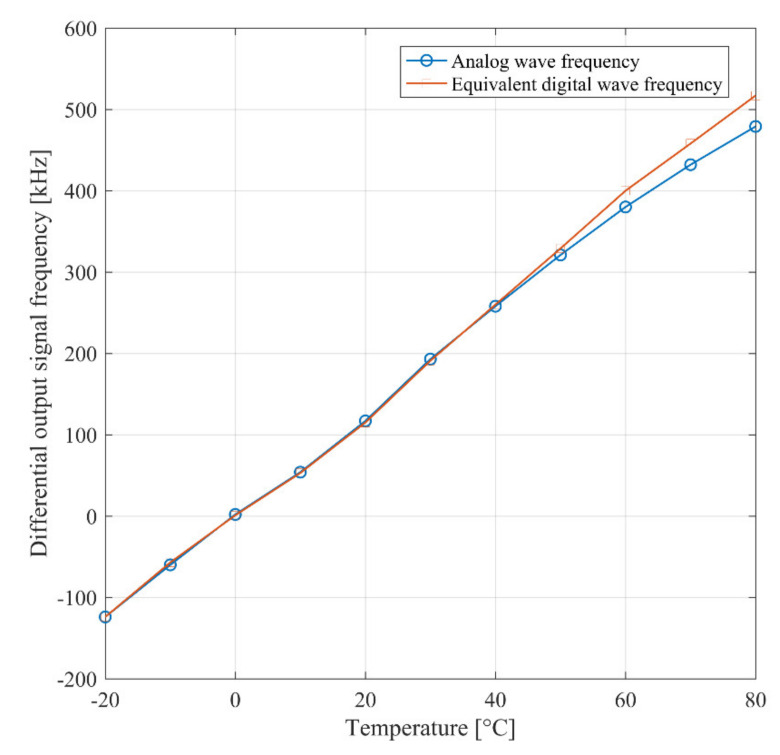
Measured Schmitt trigger output signal frequency versus temperature.

With the purpose of also demonstrating the temperature behavior expressed in the second section, measurements inside a controlled oven were executed, whose results are shown in Figure 13. The measured curve shows a positive temperature coefficient equal to 6 kHz/°C. Although this behavior seems at first sight to contradict the results reported in Figure 6, it is coherent with the analytical evaluation reported in the Appendix A. Indeed, in experimental measurements the passivation of the reference capacitor led to a load capacitance of the reference RO higher than that of the sensing RO. Consequently, as predicted by (A4) in the Appendix A, the temperature coefficient is positive.

**Figure 14:**
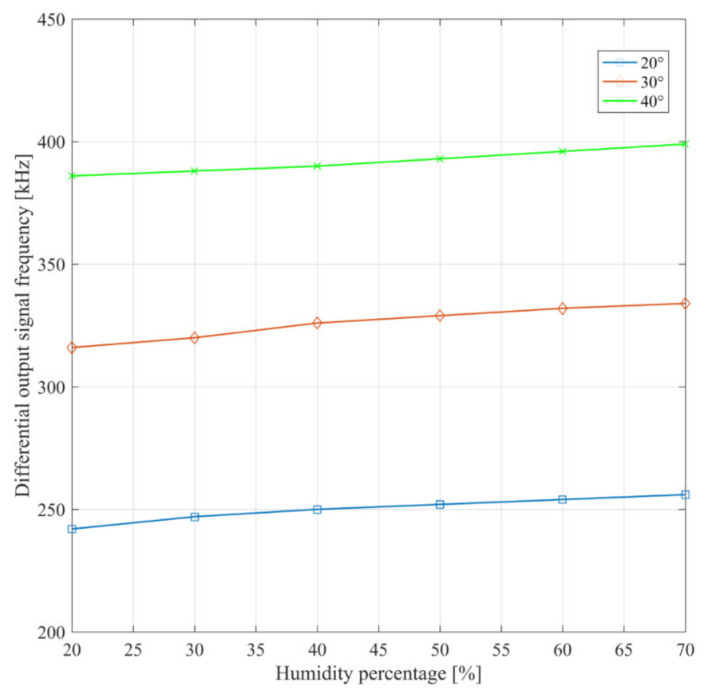
Measured Schmitt trigger output signal frequency for different humidity and temperature values.

The behavior of the system against ambient humidity variations was assessed using a test chamber where humidity changed from 20% to 70%. These measurements results are reported in Figure 14 for different values of ambient temperature, namely 20 °C, 30 °C, and 40 °C. By inspection of Figure 14, it is apparent that humidity variations produce a capacitance variation in the order of tens of aF. Moreover, they also confirm the temperature results of Figure 13.

Finally, Table 4 reports a comparison with other capacitive transducers in the literature. Among the compared solutions, the proposed sensor and [36] are the only ones providing a digital output word. Note, however, that area occupation of [36] does not include the area of the off-chip sensing MEMS capacitors. The solution proposed in [4], which targets the same application, shows a much lower resolution (0.065 aF), but at the expenses of a higher power consumption. The sensor introduced in [23] is designed for PM detection as well and shows a similar resolution, but exhibits a much higher area occupation. We can therefore conclude that the proposed sensor well compares with the state of the art, showing a good compromise between supply voltage, power consumption, area occupation, and resolution, while providing a digital output.

## 4. Conclusions

In this paper, a simple and mostly digital electronic front end for capacitive sensors is introduced. The system is designed for the detection of airborne particle matter using interdigitated planar capacitors implemented on the top metal of the die. Preliminary static experimental measurements show that the proposed system can detect talcum particles with a diameter in the order of 7 mm. The proposed circuit is just a part of the overall detecting system in which several capacitive electrodes are exploited in the detecting channel in order to enable in-flow detection. This system is currently under development.

## Figures and Tables

**Table 1:** Design parameters.

	Parameter	Value
ROs	(*W*/*L*)_PMOS_	54.8 μm/1 μm
	(*W*/*L*)_NMOS_	20 μm/1 μm
	*C_S_*_0_	330 fF
	*C_PAR_*	548 + 202 fF
	*C_TRIM_*	112.4 fF
	*N*	3
Mixer + filter	(*W*/*L*)_PMOS_	5.4 μm/0.2 μm
	(*W*/*L*)_NMOS_	2/0.2
	*R*	2 MΩ
	*C*	100 fF
Trigger	(*W*/*L*)_M1_	8.1 μm/0.13 μm
	(*W*/*L*)_M2_	0.81 μm/0.13 μm
	(*W*/*L*)_M3_	3 μm/0.13 μm
	(*W*/*L*)_M4_	0.3 μm/0.13 μm
Counter	*K*	8
Divider	*M*	32,768

**Table 2:** Corner analysis.

		TT	FF	FS	SF	SS
−40 °C	*f_ref_* (MHz)	89.62	89.02	89.62	89.6	90.19
	Δ*f* (kHz)	67.8	66.22	67.8	67.82	69.27
27 °C	*f_ref_* (MHz)	79.97	79.59	79.96	79.972	80.32
	Δ*f* (kHz)	59.07	57.89	59.05	59.07	60.35
80°	*f_ref_* (MHz)	74.46	74.217	74.46	74.46	74.71
	Δ*f* (kHz)	54.33	53.33	54.33	54.29	55.318

**Table 3:** Comparison of digital output with measured frequency variation

Trigger Output (kHz)	Counter (HEX Value)	Δ*f* (kHz)	*σ* (kHz)	Resolution * (aF)
340	H90	345	3.1	153
326	H88	326.4	3.1	153
310	H81	309.6	3.1	153
295	H7A	292.8	3	150
280	H74	278.4	3.2	159
267	H70	268.8	3.1	159
255	H6B	256.8	3.2	159
250	H66	244.8	3.3	165
235	H62	235.2	2.7	135
229	H61	232.8	2.7	135

* 3*σ* resolution.

**Table 4:** Performance summary and comparison with the state of the art.

Reference	Topology	Tech.(nm)	Supply Volt. (V)	Power (mW)	Meas. Time (ms)	Area (mm^2^)	Resolution (aF)	Application
[4]	C2V lock-in	350	3.3	84	2.5	6 ^a^	0.065	Airborne particle detector
[15]	C2V	180	1.8	0.35	0.00526	0.15	54	Differential MEMS
[23]	CBCM	800	–	–	0.02	14.62 ^a^	50 ^b^	Airborne particle detector
[27]	C2I	65	2.5	0.22	0.002	0.03	800 ^b^	Differential cap. sensor
[33]	C2F RO	350	3.3	8	1400	9 ^a^	14.4 ^b^	Lab-on-chip cell proliferation
[34]	C2T	320	3	0.084	0.385	0.52	800	Differential cap. sensor
[36]	C2D	130	1.5	0.22	20	0.317	5.4	Differential MEMS
This Work	C2F RO	130	1.2	1.316	0.416	0.12 ^a^	165	Airborne particle detector

^a^ area occupation of both front end and sensing electrodes. ^b^ 1-σ resolution.

## Data Availability

Data sharing is not applicable to this article.

## Appendix A

A first-order approximated model that considers temperature variations for the sensing and reference oscillation frequency expressed by (1) and (2) is given by

(A1) $$f_{s}(T)=\frac{2\left[I_{avg,s}\left(T_{0}\right)+\alpha \left(T-T_{0}\right)\right]}{V_{DD,s}\left(NC_{load,s}+\sum_{i=1}^{N}\Delta C_{i}\right)}$$

(A2) $$f_{ref}(T)=\frac{2\left[I_{avg,ref}\left(T_{0}\right)+\alpha \left(T-T_{0}\right)\right]}{NV_{DD,ref}C_{load,ref}}$$

where *α* is the temperature coefficient of the average current (Coefficient *α* takes into account the temperature dependence of the current due to the mobility and the threshold voltage), equal from simulations to −0.4 µA/°C for both *I_avg,s_* and *I_avg,ref_*, *T_0_* is the room temperature and *I*(*T*_0_) is the room temperature current. Equations (A1) and (A2) are written in the more general case in which the load capacitance and the supply voltage is different for the sensing and reference ROs. It should be noted that *V_DD,s_* and *V_DD,ref_* can be exploited as an additional knob to reduce the output offset frequency due to process and mismatch variations.

By deriving Equation (A2), with respect to the temperature and neglecting the temperature coefficient of *V_DD,ref_*, the frequency temperature coefficient is obtained as

(A3) $$\beta_{f_{ref}}=\frac{df_{ref}(T)}{dT}=\frac{2\alpha }{NV_{DD,ref}C_{load,ref}}$$

which yields *β* = 117 kHz/°C, that is about 7% lower than the simulated value reported in Figure 6 (126 kHz/°C).

The temperature coefficient of the frequency difference can be obtained by substituting Equations (A1) and (A2) in Equation (2) and evaluating the first derivative respect to temperature, yielding

(A4) $$\beta_{\Delta f}=\frac{d\Delta f(T)}{dT}=2\alpha \left[\frac{1}{V_{DD,s}\left(NC_{load,s}+\sum_{i=1}^{N}\Delta C_{i}\right)}-\frac{1}{NV_{DD,ref}C_{load,ref}}\right]$$

Assuming *V_DD,s_* = *V_DD,ref_* = *V_DD_* and neglecting the second order term we get

(A5) $$\beta_{\Delta f}=\frac{d\Delta f(T)}{dT}\approx \frac{2\alpha \sum_{i=1}^{N}\Delta C_{i}}{V_{DD}{\left(NC_{load,ref}\right)}^{2}}$$

The numerical value predicted by Equation (A5), equal to 92.5 Hz/°C, which is 14% lower than the simulated value in Figure 6 (108 Hz/°C).
